# Magnetic-Map-Matching-Aided Pedestrian Navigation Using Outlier Mitigation Based on Multiple Sensors and Roughness Weighting

**DOI:** 10.3390/s19214782

**Published:** 2019-11-03

**Authors:** Yong Hun Kim, Min Jun Choi, Eung Ju Kim, Jin Woo Song

**Affiliations:** Department of Software Convergence, Sejong University, 209 Neungdong-ro, Gwangjin-gu, Seoul 05006, Korea; yhkim@sju.ac.kr (Y.H.K.); cmj1512@sju.ac.kr (M.J.C.); garon0927@sju.ac.kr (E.J.K.)

**Keywords:** magnetic field map matching, roughness weighting, importance sampling, indoor navigation

## Abstract

This research proposes an algorithm that improves the position accuracy of indoor pedestrian dead reckoning, by compensating the position error with a magnetic field map-matching technique, using multiple magnetic sensors and an outlier mitigation technique based on roughness weighting factors. Since pedestrian dead reckoning using a zero velocity update (ZUPT) does not use position measurements but zero velocity measurements in a stance phase, the position error cannot be compensated, which results in the divergence of the position error. Therefore, more accurate pedestrian dead reckoning is achievable when the position measurements are used for position error compensation. Unfortunately, the position information cannot be easily obtained for indoor navigation, unlike in outdoor navigation cases. In this paper, we propose a method to determine the position based on the magnetic field map matching by using the importance sampling method and multiple magnetic sensors. The proposed method does not simply integrate multiple sensors but uses the normalization and roughness weighting method for outlier mitigation. To implement the indoor pedestrian navigation algorithm more accurately than in existing indoor pedestrian navigation, a 15th-order error model and an importance-sampling extended Kalman filter was utilized to correct the error of the map-matching-aided pedestrian dead reckoning (MAPDR). To verify the performance of the proposed indoor MAPDR algorithm, many experiments were conducted and compared with conventional pedestrian dead reckoning. The experimental results show that the proposed magnetic field MAPDR algorithm provides clear performance improvement in all indoor environments.

## 1. Introduction

A position is an important piece of information for personal navigation, and various methods have been used to obtain the position. In particular, finding a person’s indoor position is important because it can be used for workers with special duties, to improve work efficiency. In addition, a personal navigation system can be applied to the aged and patients to sustain their security. Analyses of the positions of pedestrians can also be used in various indoor applications, such as location-based services for marketing.

In the case of the outdoors, the location information can be obtained by using the global navigation satellite system (GNSS), as well as other precision-navigation technologies that fuse GNSS and sensor data with map information. However, there is a limitation in using accurate position information for indoor cases because the GNSS signal is not available in general. Therefore, many research studies have been conducted to obtain accurate position information indoors, especially for robotics and personal navigation.

Studies based on various sensors have been conducted to find the position indoors, where GNSS signals cannot be received. The light detection and ranging (LiDAR) sensor is a widely used sensor for finding the position in a room, and its position-detection performance has been proven when it is fused with other sensors using various filters [1,2]. In particular, map-based position detection using simultaneous localization and mapping (SLAM) has the advantage of finding the absolute position [3]. However, the LiDAR sensor is mainly used for unmanned ground vehicles (UGVs), robotics, or unmanned systems, rather than pedestrians, because it is difficult to attach sensors to the human body efficiently.

Another approach to detecting indoor locations is based on various wireless signals such, as Wi-Fi, Bluetooth, and ultra-wideband (UWB) in a wireless local area network (WLAN) [4,5]. However, this method has the disadvantage of high cost because it requires the infrastructure for the entire building for which the position information is requested. Although it can measure the absolute position, the location accuracy is not sufficient in some cases.

Another way to detect the position indoors is by using pedestrian dead reckoning (PDR) with an inertial measurement unit (IMU). PDR is a technique for estimating the position of pedestrians by attaching an IMU and analyzing the movement characteristics of pedestrians. It has the advantages of a high output rate and dynamic performance. The performance of PDR-based navigation was verified by the International Indoor Positioning and Indoor Navigation (IPIN) competitions held in 2015, 2016, and 2017 [6].

The performance of the PDR-based navigation system was superior to other algorithms in most situations. Nevertheless, it has fatal shortcomings in the divergence of the position error with time. Thus, various research studies have been carried out for mitigating divergent position errors [7,8]. Studies for detecting the stance phase accurately [9,10,11] have been carried out. Meaningful research studies have been conducted to improve the accuracy of headings by attaching additional sensors to the foot [12,13,14,15,16]. 

However, indoor-position-detection methods based only on the PDR algorithm cannot avoid position errors, fundamentally because they do not use the measurements of absolute position. As a result, an additional sensor that can compensate for position errors should be used to construct a highly accurate personal navigation system. Research studies on the integration of the PDR and WLAN-based positions have been studied for achieving accurate positions [17,18].

A position detection technique using magnetic sensors has also been developed. This is a method for detecting the position based on the deformed magnetic field anomaly measured in the building and comparing it with the measured value [19,20,21,22,23,24,25]. Indoor position detection methods using magnetic sensors have the advantages that the absolute position is measured without any additional information and that no infrastructure is necessary compared with WLAN-based positioning methods. Because a deformed magnetic field mainly depends on the structure of a building, it can be used to determine a position. However, the deformed sensor data have some ambiguities because the structure of a building is, in general, formal and uniform. Thus, personal navigation algorithms that integrate PDR and geomagnetic information are currently being studied [26,27]. In particular, recent researches have conducted indoor-location estimation by using PDR and magnetic sensor, using a smartphone [28,29].

However, the conventional position detecting methods use only the algorithms that simply compare the magnetic fields and sensor data, which cannot avoid the error caused by the ambiguity of the magnetic field similarity.

To overcome this problem, we propose an ambiguity mitigation algorithm based on multiple-sensors and roughness weighting, in order to reduce outliers that are caused by similar magnetic fields. By using multiple sensors, the enhanced distinguishability is achievable. In this study, two magnetic sensors, which were mounted on a foot and around a waist, were used, and the experimental configuration is described in detail in Section 5. In particular, showed that the accuracy of matching is improved when the roughness concept and weighting factor, according to the roughness, are employed. By employing the roughness weighting, the sensor data having more roughness can be more emphasized, which results in outlier mitigation. We also use an important sampling-based position-estimation method that considers all ambiguous positions [30,31,32]. In this study, we propose the restricted boundary resampling method to improve map-matching performance. The boundary for resampling is determined by the pedestrian’s stride and its stochastic model. The proposed method robustly and precisely determines the candidate indoor position of a pedestrian. Finally, the estimated position is used as a measurement to update the Kalman filter and to compensate the sensor and navigation errors of the PDR, which results in the improvement of indoor PDR performance. As the position accuracy improves, the proposed method can also be used in many application areas, such as a smart factory, a hospital, a sanitarium, and other social infrastructures. 

This paper is organized as follows. In Section 2, the conventional stance phase detection method for the zero-velocity update (ZUPT), the PDR based on the ZUPT, and the extended Kalman filter (EKF) are described. In Section 3, the position detection method based on the magnetic field map and outlier mitigation method are presented. The position-error-reduction algorithm based on importance sampling and restricted boundary resampling technique is also presented. Section 4 describes how to construct an indoor pedestrian navigation algorithm with the measurements as ZUPT and estimated position. In Section 5, the results of the experiments are presented to verify the performance of the proposed method. Finally, we conclude with some remarks and further research topics.

## 2. Pedestrian Dead Reckoning Using Zero Velocity Update

The navigation algorithms based on PDR basically use the integration of acceleration and angular rate for position and orientation calculations. During the integration process, the bias errors of the accelerometer and gyroscope induce orientation and velocity error, diverging with time, which results in the divergence of position with time. To suppress the velocity divergence, zero velocity information during the stance phase is used.

The walking gait characteristics of a person are divided into a stance phase and swing phase. During the stance phase, the foot touches the ground, and in the swing phase, the foot moves away from the ground. Even though the velocity of the body during walking is not zero, the foot velocity at the stance phase is zero. The ZUPT method uses the fact that the foot velocity is zero if it is determined to be in the stance phase, and an IMU attached to the foot measures the sensor data to determine the stance phase. This method can prevent velocity divergence over time. Therefore, it is important to detect the exact stance phase, and various methods for detecting the stance phase have been studied [12].

**Figure 1:**
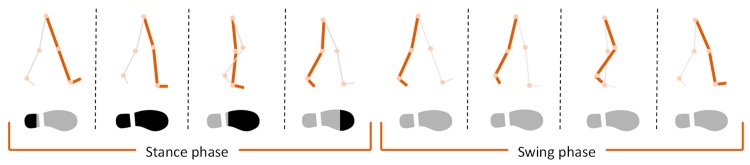
Gait characteristics and stance-phase detection.

Among them, the stance-phase detection method considering the energy, product, and sum (EPS) of the acceleration and the energy of the angular rate can detect the stance phase in various situations more efficiently because it can exclude an excessive acceleration or rotating rate during the heel strike (first step in Figure 1) and push-off phase (fourth step in Figure 1). For stance phase detection and stride estimation, it has high reliability, with an error of less than 1% [8,10]. 

The energy $E_{k}$, product $P_{k}$, and sum $S_{k}$ of the acceleration, and the energy of angular rate $w_{k}$ at time step k, can be defined as follows:

(1) $$E_{k}=\sqrt{a_{x}{}^{2}\left(k\right)+a_{z}{}^{2}\left(k\right)},$$

(2) $$P_{k}=a_{x}\left(k\right)a_{z}\left(k\right),$$

(3) $$S_{k}=a_{x}\left(k\right)+a_{z}\left(k\right),$$

(4) $$w_{k}=\sqrt{w_{x}{}^{2}\left(k\right)+w_{y}{}^{2}\left(k\right)},$$

where $a_{x}\left(k\right),\;a_{z}\left(k\right),\;w_{x}\left(k\right)$, and $w_{y}\left(k\right)$ are the acceleration along the x and *y* axes, and the angular rate along the *x* and *y* axes, respectively. To detect the stance phase, the variance of 15 sample data for each of them is compared with a threshold as follows:

(5) $$C_{1}=\{\begin{array}{cc} 1 & \mathrm{Var}\left(E_{k-14}:E_{k}\right)<th_{E}\\ 0 & \mathrm{otherwise} \end{array},$$

(6) $$C_{2}=\{\begin{array}{cc} 1 & \mathrm{Var}\left(P_{k-14}:P_{k}\right)<th_{P}\\ 0 & \mathrm{otherwise} \end{array},$$

(7) $$C_{3}=\{\begin{array}{cc} 1 & \mathrm{Var}\left(S_{k-14}:S_{k}\right)<th_{S}\\ 0 & \mathrm{otherwise} \end{array},$$

(8) $$C_{4}=\{\begin{array}{cc} 1 & \mathrm{Var}\left(w_{k-14}:w_{k}\right)<th_{w1}\\ 0 & \mathrm{otherwise} \end{array},$$

(9) $$C_{5}=\{\begin{array}{cc} 1 & \left|w_{k}\right|<th_{w2}\\ 0 & \mathrm{otherwise} \end{array}.$$

When all conditions, $C_{1}$, $C_{2}$, $C_{3}$, $C_{4}$, $\mathrm{and}\;C_{5}$, are satisfied and become 1, a stance phase is declared by the algorithm.

In pedestrian dead reckoning, the velocity and attitude error and sensor error must be corrected in order to calculate the exact position. In most cases, an EKF is used for error compensation because the error model of the pedestrian dead reckoning system is nonlinear. Therefore, the 12th-order EKF is widely used, and the navigation algorithm is implemented based on the EKF with ZUPT [33,34]. The EKF estimates the velocity error, attitude error, gyroscope bias, and accelerometer bias. However, ZUPT-based PDR diverges with time because it does not use position measurements. Therefore, in this study, the 15th-order error model is constructed and used for EKF by integrating the result, using the map matching, which contributes to the non-diverging and accurate position. The error model, the measurements, and the algorithms to which the position information is added are discussed in Section 4. 

## 3. Magnetic Map Matching Using Multiple Sensors

### 3.1. Anomaly of Indoor Magnetic Field

The original purpose of the magnetic sensor was to measure the geomagnetic field of the earth and find the magnetic north. However, the magnetic field is easily distorted by metal or magnetic substances, which means that the magnetic sensor is very sensitive to the surrounding environment, and it is not easy to measure the correct geomagnetic field. The magnetic field distortion is classified into the hard-iron effect and the soft-iron effect, according to the distorting characteristics of the magnetic field. The hard-iron effect is a phenomenon in which the magnetic field is distorted by other magnetic substances. The soft-iron effect is a phenomenon in which the magnetic field is distorted by metallic material. 

In particular, since the building structure is composed of various steel structures and iron H beams, the magnetic field inside the building is easily distorted, and the distortion varies according to the location. Thus, the location in a building can be determined by detecting the distorted magnetic field. The more the magnetic field is distorted, the more accurately the location can be determined. On the other hand, the severe magnetic distortion prevents us from finding the magnetic north correctly. Therefore, it is very challenging to achieve the purpose of measuring the magnetic heading and position mapping simultaneously. 

**Figure 2:**
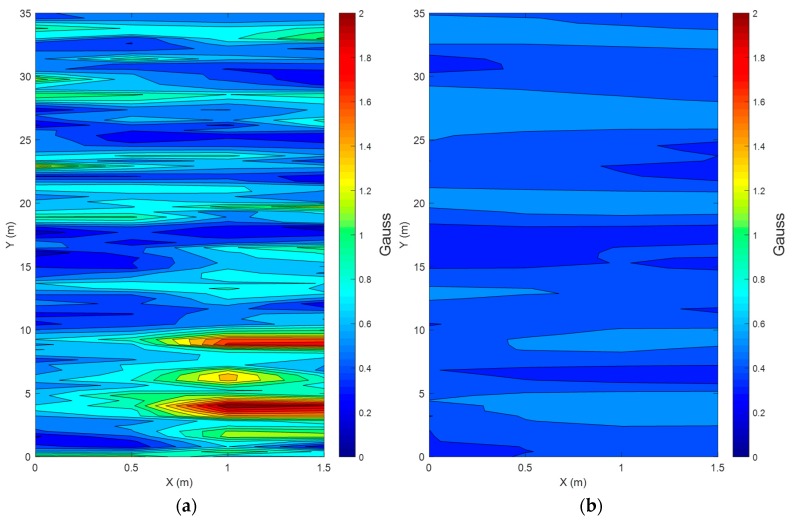
Magnetic anomaly maps in the corridor ((**a**) is magnetic anomaly around the foot, and (**b**) is magnetic anomaly around the waist).

Figure 2 shows the magnetic anomaly maps measured in the corridor after attaching a magnetic sensor to the foot and the waist. The anomaly maps present the distortion of the magnetic field, which is affected by the hard-iron effect and soft-iron effect. Magnetic anomaly refers just to the variations of the magnetic-field absolute values, which are norms of 3D magnetic vectors. In particular, the sensor attached to the foot suffers from large distortions owing to the iron structure inside the building because it is close to the building. However, the results show that each location in the corridor has its own identical magnetic field to some extent. The anomaly map of the magnetic sensor attached to the waist shows that the magnetic field is influenced by the magnetic disturbance, but the distortion is less serious, which means the magnetic heading information calculated by the sensor is less vulnerable.

Magnetic field distortions in a building are unique characteristics that depend on the size and location of the steel structures in the building, and the steel structure inside the building does not change after the building is built [33]. Thus, the magnetic map-matching that uses magnetic distortion can be applied for determining the location. Moreover, the magnetic heading can also be obtained more accurately by adopting multiple three-axis magnetic sensors attached to different parts of the body.

### 3.2. Outlier Mitigation Technique Based on Multiple Magnetic Sensors, Roughness Weighting, and Normalization

The magnetic map-matching method using multiple sensors has the advantage that the absolute position and relatively accurate heading are detectable. When using multiple sensors, it is also expected that the mapping redundancy and position ambiguity can be restrained because additional information is used. For magnetic map matching, in general, the norm of the measured magnetic field is compared with that of the stored norm according to position [33]. Since the characteristics of magnetic distortion are different according to the sensor configuration, the additional information has significant meaning. Instead of using one-dimensional data for comparison, two-dimensional information can be used for comparison, which enhances the possibility of the correct map matching. 

When a pedestrian is walking in a room where the map is built, the magnetic data from the sensor are compared with the entire set of data in the magnetic map at every stance phase. By checking the likelihood, the position can be determined. To compare the magnetic field map and measurements, the cost function is defined by the mean square deviation method [33].

When a single sensor is used for a magnetic-field map matching, only a magnitude comparison is performed. Therefore, several candidate positions may have a similar magnetic-field magnitude, which means many outliers may exist. The outliers exert bad influences on position accuracy because they increase position redundancy and ambiguity. 

However, when multiple magnetic sensors are employed, more than two magnetic field norms, which compose a vector, are used for comparison. In this case, the cost function must be modified as follows:

(10) $$J\left(i,j\right)=\|M_{s}\left(p_{k}\right)-M_{m}\left(p_{m}\left(i,j\right)\right)\|,$$

(11) $${\hat{p}}_{m,k}=\underset{p_{m}\left(i,j\right)}{\arg\;\min}J\left(i,j\right),$$

where $M_{s}\left(p_{k}\right)$ is the vector whose components are the norms of the magnetic field from the multiple sensors at current position $p_{k}\in {\mathbb{R}}^{3}$, and $M_{m}\left(p_{m}\left(i,j\right)\right)={\left[\begin{array}{cc} M_{f}\left(p_{m}\left(i,j\right)\right) & M_{w}\left(p_{m}\left(i,j\right)\right) \end{array}\right]}^{T}$ is the pre-stored magnetic norm vector pre-acquired at candidate grid point $p_{m}\left(i,j\right)\in {\mathbb{R}}^{3}$. Here, $M_{f}\left(p_{m}\left(i,j\right)\right)$ and $M_{w}\left(p_{m}\left(i,j\right)\right)$ represent magnetic norms obtained from the foot-mounted sensor and waist-mounted sensor, respectively, and $\left(i,j\right)\in S_{M\times N}$ is a grid map index vector, where $S_{M\times N}$ is a 2D grid index domain with the size of M×N. Since the floor height is used for height in the 3D magnetic map, only the 2D magnetic anomaly map is built using 3D magnetic sensor and considered for map matching. The position on the grid map where the cost function (J) is minimized becomes the candidate current position (${\hat{p}}_{m,k}$).

If multiple sensors are affected by magnetic disturbance differently, the vector may have a unique direction, which implies every grid point may have its uniqueness. When each magnetic-map grid point has unique values, the outlier can be eliminated easily, and magnetic map matching will provide more accurate results. 

In this study, this advantageous feature of using multiple sensors is validated by employing the roughness concept, which quantifies the degree of the magnetic-field distortion. The uniqueness of each datum on the magnetic map can be qualified by comparing the data with near data. Therefore, the roughness index at $p_{m}\left(i,j\right)$ on $S_{M\times N}$ is defined as follows:

(12) $$R_{u}\left(p_{m}\left(i,j\right)\right)=\frac{1}{MN-1}\left\{\overset{\;}{\underset{\left(x,y\right)\in S_{M\times N}-\left\{\left(i,j\right)\right\}}{\sum }}\frac{\left\|M_{m}\left(p_{m}\left(i,j\right)\right)-M_{m}\left(p_{m}\left(x,y\right)\right)\right\|}{\sqrt{{\left(i-x\right)}^{2}+{\left(j-y\right)}^{2}}}\right\}.$$

Here, the denominator is added to impose the weightings on data close to the test point $\left(p_{m}\left(i,j\right)\right)$). Even though the roughness is a relative quantity, the large roughness index implies the test point can be locally well characterized, which contributes to the reduction of position ambiguity. 

**Figure 3:**
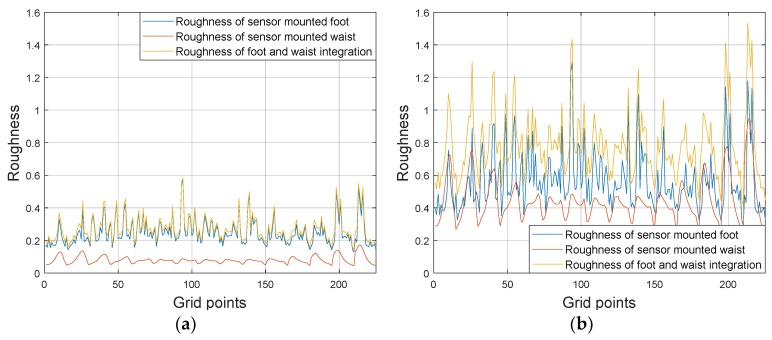
Magnetic-map roughness ((**a**) is for simple integration case and (**b**) is for normalized integration case with roughness weighting factors).

Using multiple sensors enhances the roughness because vector information is used in comparing map data and measurements, as shown in Figure 3a. However, the improvement might be limited if one piece of information has much larger roughness. In this study, sensors are attached to the foot and waist, respectively. In this case, the roughness according to the foot-mounted sensor is much larger than that from the waist-mounted sensor, which restricts roughness improvement. Moreover, the roughness indices obtained from different sensors need to be compared with each other carefully because they are relative quantity. 

To overcome this issue, the normalized magnetic fields are devised for the roughness test and map matching, and the roughness index is modified as follows:

(13) $$R_{u}\left(p_{m}\left(i,j\right)\right)=\frac{1}{MN-1}\left\{\overset{\;}{\underset{\left(x,y\right)\in S_{M\times N}-\left\{\left(i,j\right)\right\}}{\sum }}\frac{\|{\tilde{M}}_{m}\left(p_{m}\left(i,j\right)\right)-{\tilde{M}}_{m}\left(p_{m}\left(x,y\right))\right\|}{\sqrt{{\left(i-x\right)}^{2}+{\left(j-y\right)}^{2}}}\right\}.$$

Here,

(14) $${\tilde{M}}_{m}\left(p_{m}\left(i,j\right)\right)=\left[\begin{array}{cc} \alpha \cdot \sigma_{M_{f}}^{-1} & 0\\ 0 & \beta \cdot \sigma_{M_{w}}^{-1} \end{array}\right]\left[\begin{array}{c} M_{f}\left(p_{m}\left(i,j\right)\right)\\ M_{w}\left(p_{m}\left(i,j\right)\right) \end{array}\right]=N_{g}\cdot M_{m}\left(p_{m}\left(i,j\right)\right),$$

$\sigma_{M_{f}}$ and $\sigma_{M_{w}}$ are the standard deviations of each magnetic field data, and α and β are tuning factors which satisfy α+β=1. $N_{g}$ is a weighting matrix consisting of standard deviation and tuning factors. In this study, a simple normalization matrix is not used, but tuning factors, which are roughness weightings, and they are multiplied to the normalizing factors. If the measured magnetic field were randomly distributed, the roughness weighting factors might not necessarily need to be multiplied. However, the measured data are not randomly distributed, but affected by the building structure and geomagnetic field. Thus, we employed the roughness weighting factors for equalizing their effects on each magnetic datum. The weighting factor is dependent on environments and decided via experiments during map building. Now, the modified cost function and modified map-matching method using the mean square deviation can be defined as follows:

(15) $$\tilde{J}\left(i,j\right)=\|N_{g}\cdot M_{s}\left(p_{k}\right)-{\tilde{M}}_{m}\left(p_{m}\left(i,j\right)\right)\|,$$

(16) $${\hat{p}}_{m,k}=\underset{p_{m}\left(i,j\right)}{\arg\;\min}\tilde{J}\left(i,j\right).$$

By engaging the normalized magnetic data and roughness weighting factors, the roughness can be improved, as shown in Figure 3b. 

To validate the roughness concept, we experimented several times in various indoor environments. The experimental results are summarized in Table 1. The results show that the roughness is improved in various cases when multiple sensors are employed, and, eventually, the position redundancy and ambiguity can be improved, as well, which implies outliers can be reduced by using multiple sensors and normalized magnetic data.

**Figure 4:**
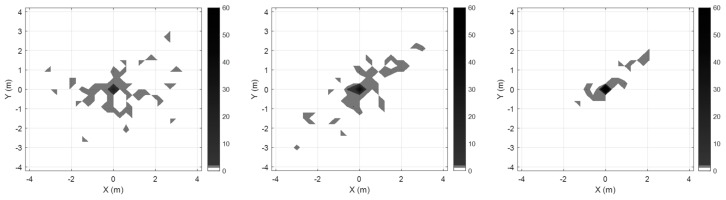
Position map-matching errors and outlier distributions for three cases ((**Left**) foot-mounted sensor, (**Middle**) waist-mounted sensor, and (**Right**) multiple sensor case with simple integration).

**Figure 5:**
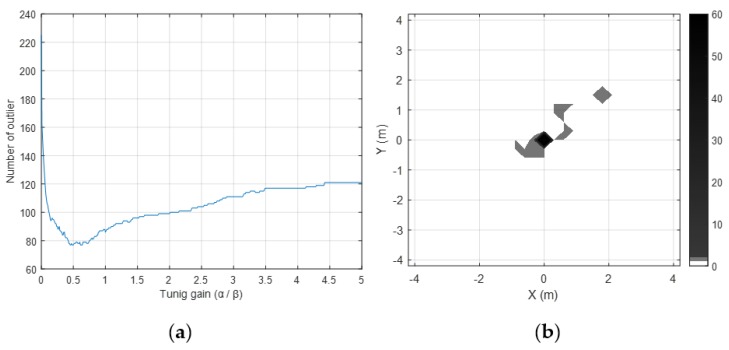
(**a**) Represents the number of outliers according to the tuning gain. (**b**) Shows the outlier when the roughness weighting factors are applied.

Using the normalization with roughness weighting and multiple sensors, the outlier can be mitigated, as well. To evaluate the outlier mitigation performance of the proposed method, we also performed several experiments. Figure 4 shows map-matching errors and outlier distributions for three cases: foot-mounted sensor case, waist-mounted sensor case, and multiple sensor case without normalization ($N_{g}=I_{2\times 2})$. Experimental results show that the error is reduced, and outliers are mitigated more effectively when multiple sensors are used rather than when a single sensor is used. The result of the multi-sensor case with normalization ($N_{g}$) is shown in Figure 5. Figure 5a shows that the minimum outlier is achievable when α/β=0.5, that is, when $\alpha =\frac{1}{3}$ and $\beta =\frac{2}{3}$. The position error and outlier distribution are shown in Figure 5b, which shows that the outliers are more mitigated when multiple sensors with normalization factors are used rather than multiple sensors are used simply. The proposed outlier mitigation technique is verified through several experiments, whose results are summarized in Table 2. The results show that the outliers are reduced by the maximum of 62%, owing to the proposed technique, and overall position error is reduced by 40–86%.

### 3.3. Improvement of Position-Measurement Quality Using Importance Sampling

Although the use of multiple sensors and normalization methods can improve the map-matching accuracy, improper detection cannot be avoided completely because of the unavoidable ambiguity caused by uniform and repeated building structures. For example, iron doors spaced at equal intervals along a corridor induce similar magnetic distortions at a different position, which may result in an inaccurate map-matching solution.

To solve this problem, a candidate boundary, which is based on the simple probabilistic model, can be employed to determine the candidate positions for map matching. However, in this case, it is difficult to form an optimal boundary because the limited candidate boundary may result in the local minim of the cost function. For this reason, this study uses stride-based probabilistic sampling to create optimal boundaries and uses importance sampling to determine the proper position within the boundary. Importance sampling is a kind of Monte Carlo method that weights the samples determined through the sampling step and obtains the final estimate. Importance sampling can solve the problem by simply calculating the position with the cost function. The sampling process makes it possible to prevent a large position error due to the repeated magnetic patterns because it puts the weightings on the samples with importance.

For importance sampling, the candidate boundary for collecting samples should be determined first. In this research, it is assumed that the candidate positions are not far from the previous position. The boundary is selected efficiently by calculating the previous strides and smoothing them. To calculate the diameter of the boundary, the smoothing filter is employed as follows:

(17) $$d_{k}=0.4\cdot d_{k-1}+0.6\cdot \|{\hat{p}}_{k-2}^{+}-{\hat{p}}_{k-1}^{+}\|,$$

where ${\hat{p}}_{k}^{+}$ is a position estimate at time k, which is updated at the stance phase. To prevent local minima caused by the sampling within the limited boundary, the diameter of the candidate boundary for magnetic map matching is expanded to six times larger than $d_{k}$.

Simultaneously, position candidates that have high likelihood but locate abnormally far away from the previous position should be ignored, so that a large position error is prevented. To do this, the importance sampling method is employed. Within the candidate boundary, the appropriate L-sampled positions on the grid map, whose cost functions, $\tilde{J}$, are all small, are extracted. Instead of using the position with a minimum cost function, L-sampled positions are all used for fixing the position by applying importance weightings. 

The sampled positions, $p_{m}^{s}\left(i,j\right)$, are assumed to be random variables with Gaussian normal probability density function (PDF), and their variances are determined by their importance weightings. In this research, we define the importance weighting at the sampled position, $p_{m}^{s}\left(i,j\right)$, as the function of the cost function defined in Equation (15) and distance from the prior position estimate, ${\hat{p}}_{k}^{-}$, obtained by the PDR. Therefore, the importance weighting or variance of the sampled position, $p_{m}^{s}\left(i,j\right)$, is defined as follows:

(18) $$\sigma_{i,j}=\tilde{J}\cdot \|{\hat{p}}_{k}^{-}-E\left(p_{m}^{s}\left(i,j\right))\right\|.$$

Using the importance weightings and variances, the weighted PDF for the sampled position can be obtained as follows:

(19) $$\tilde{w}\left(p_{m}^{s}\right)\sim \;N\left(E\left(p_{m}^{s}\left(i,j\right)\right),\sigma_{i,j}\right)=N\left(p_{m}\left(i,j\right),\sigma_{i,j}\right).$$

When L samples are extracted, the PDF of a sample can be normalized as follows:

(20) $$w\left(p_{m}^{s}\right)=\frac{\tilde{w}\left(p_{m}^{s}\right)}{\sum_{}^{L}\tilde{w}\left(p_{m}^{s}\right)}$$

Because the defined weighted PDF is normalized, the position can be chosen as the sum of the samples to which importance weightings are applied as follows:

(21) $${\hat{p}}_{m,k}=\underset{\left(i,j\right)\in S_{L}}{\sum }E\left(p_{m}^{s}\left(i,j\right)\right)w\left(p_{m}^{s}\right)=\underset{\left(i,j\right)\in S_{L}}{\sum }p_{m}\left(i,j\right)w\left(p_{m}^{s}\right),$$

which becomes the final map-matched position and also the position measurement for a Kalman filter. Here, $S_{L}$ is the set of all sampled position indices. For determining the quality of the measurement, the covariance $R_{p}$ of ${\hat{p}}_{m,k}$ should be calculated as follows:

(22) $$R_{p}=\underset{\left(i,j\right)\in S_{L}}{\sum }\left(E\left(p_{m}^{s}\left(i,j\right)\right)-{\hat{p}}_{m,k}\right){\left(E\left(p_{m}^{s}\left(i,j\right)\right)-{\hat{p}}_{m,k}\right)}^{T}w\left(p_{m}^{s}\right).$$

**Figure 6:**
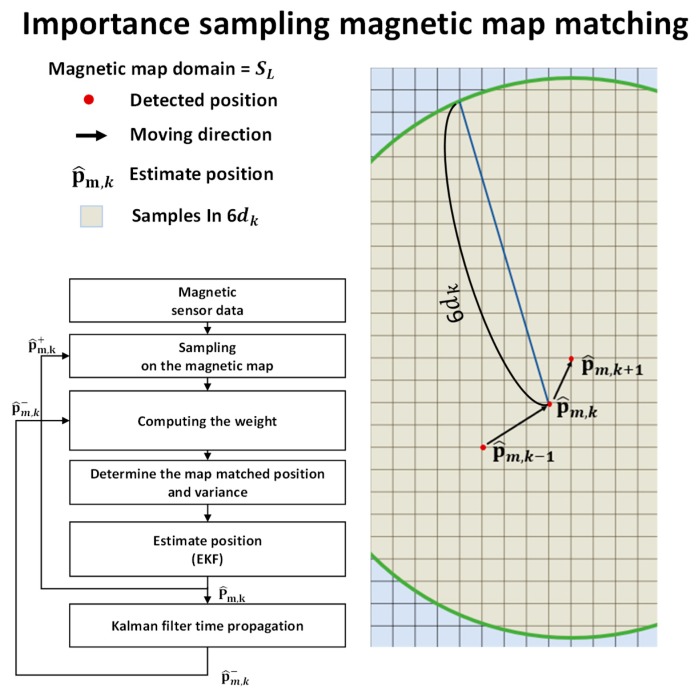
Concept of magnetic map-matching algorithm using importance sampling.

Figure 6 shows the conceptual diagram of importance sampling, candidate boundary, and algorithm flowchart. The magnetic map-matching algorithm using importance sampling is summarized as follows:

1. Calculate the stride from the previous positions and set a candidate boundary.
2. Perform sampling within the candidate boundary.
3. Compute the importance weightings (or cost functions) corresponding to each sampled position.
4. Using the roughness weightings and normalization, find the normalized PDF for each sampled position.
5. Determine the map-matched position and its covariance.
6. Use the position and covariance as a new measurement for the measurement update of the Kalman filter.

## 4. Magnetic-Map-Matching-Aided Pedestrian Dead Reckoning

In Section 3, it is verified that the number of outliers is reduced, and the magnetic-map-matching accuracy is improved by employing multiple magnetic sensors and proposed mitigation techniques. This section describes combining PDR and magnetic map matching. By integrating them, it is expected that the positioning accuracy will be improved even when a magnetic map does not cover all the areas. 

In this study, map-matching-aided pedestrian dead reckoning (MAPDR) was constructed. To estimate and compensate for the navigation errors, a 15th-order EKF is used. The error state vector is given by the following equation:

(23) $$x={\left[\delta p^{T}\;\delta v^{T}\;\delta \phi^{T}\;b_{g}^{T}\;b_{a}^{T}\right]}^{T},$$

where $\delta p={\left[\delta p_{N}\;\delta p_{E}\;\delta p_{D}\right]}^{T}$, $\delta v={\left[\delta v_{N}\;\delta v_{E}\;\delta v_{D}\right]}^{T}$, and $\delta \phi ={\left[\delta \phi_{N}\;\delta \phi_{E}\;\delta \phi_{D}\right]}^{T}$ are the position error, velocity error, and attitude error in the North-East-Down (NED) coordinate system, respectively. The gyro bias and acceleration bias are $b_{g}$ and $b_{a}$, respectively. Since the navigation sensors used in a PDR cannot measure the earth rate, the error models, including the position error, can be simplified as follows [34]:

(24) $$\delta \dot{p}=\delta v,$$

(25) $$\delta \dot{v}=S\delta \phi +C_{b}^{n}b_{a},$$

(26) $$\delta \dot{\phi }=-C_{b}^{n}b_{g},$$

where $C_{b}^{n}$ is a rotation matrix from the body frame to NED frame, and S is a skew-symmetric matrix corresponding to input acceleration ${\left[a_{N}\;a_{E}\;a_{D}\right]}^{T},$ which is defined as follows:

(27) $$S=\left[\begin{array}{ccc} 0 & -a_{D} & a_{E}\\ a_{D} & 0 & -a_{N}\\ -a_{E} & a_{N} & 0 \end{array}\right].$$

Thus, the 15th-order state-space-error model can be expressed as follows:

(28) $$\dot{x}=Fx+w=\left[\begin{array}{ccccc} 0_{3\times 3} & I_{3\times 3} & 0_{3\times 3} & 0_{3\times 3} & 0_{3\times 3}\\ 0_{3\times 3} & 0_{3\times 3} & S & 0_{3\times 3} & C_{b}^{n}\\ 0_{3\times 3} & 0_{3\times 3} & 0_{3\times 3} & -C_{b}^{n} & 0_{3\times 3}\\ 0_{3\times 3} & 0_{3\times 3} & 0_{3\times 3} & -\gamma_{g}I_{3\times 3} & 0_{3\times 3}\\ 0_{3\times 3} & 0_{3\times 3} & 0_{3\times 3} & 0_{3\times 3} & -\gamma_{a}I_{3\times 3} \end{array}\right]x+w,$$

where w is the input noise vector, and I is an identity matrix. The biases of the accelerometer and gyroscope sensors are assumed to be a first-order Markov process, with a large time constant to compensate the slowly varying bias drift over time. Thus, $\gamma_{g}$ and $\gamma_{a}$ are set to be very small in this model. 

In addition to the position measurements from magnetic map matching, the heading information using the magnetic sensor mounted on the waist is utilized as a measurement for preventing the divergence of the yaw angle. While ZUPT is performed repeatedly in a stance phase, the magnetic heading update and position update are carried out once in the stance phase because they are relatively less accurate than the zero velocity measurements. Therefore, two measurement models are required. One is for the position and heading measurement update at the time when the stance phase starts, which is defined as follows:

(29) $$z_{1}=H_{1}x+\nu_{1}=\left[\begin{array}{cccc} I_{3\times 3} & 0_{3\times 3} & 0_{3\times 3} & 0_{3\times 6}\\ 0_{1\times 3} & 0_{1\times 3} & \left[\begin{array}{ccc} \tan\phi_{N}\cos\phi_{D} & \tan\phi_{N}\sin\phi_{D} & -1 \end{array}\right] & 0_{1\times 6} \end{array}\;\right]x+\nu_{1}.$$

In this case, the measurement becomes $z_{1,m}={\left[\begin{array}{cc} {\hat{p}}_{m,k}^{T} & \phi_{D,m} \end{array}\right]}^{T},$ where ${\hat{p}}_{m,k}$ is obtained by importance sampling and magnetic map matching, and $\phi_{D,m}$ is a heading measurement obtained from the waist-mounted magnetic sensor. The measurement covariance matrix, $R_{1},$ for the measurements noise, $\nu_{1}$, is composed of $R_{p}$, which is automatically calculated by the importance sampling and $\sigma_{h}^{2}\;$, which represents the variance of the heading measurement. Although the magnetic field around the waist is less distorted, the heading information is not sufficiently accurate. Moreover, the position measurement can be used for the EKF, so that $\sigma_{h}^{2}$ is set to a slightly large value.

The other measurement model is for ZUPT during the stance phase, which is expressed as follows:

(30) $$z_{2}=H_{2}x+\nu_{2}=\left[\begin{array}{ccccc} 0_{3\times 3} & I_{3\times 3} & 0_{3\times 3} & 0_{3\times 3} & 0_{3\times 3} \end{array}\right]x+\nu_{2},$$

where $\nu_{2}$ is the measured noise vector modeled as white Gaussian noise with covariance, $R_{v}$. The velocity measurement is $z_{2,m}={\left[\begin{array}{ccc} 0 & 0 & 0 \end{array}\right]}^{T}$, the and velocity measurement noise covariance $R_{v}$ is usually small when the stance phase can be detected perfectly. 

**Figure 7:**
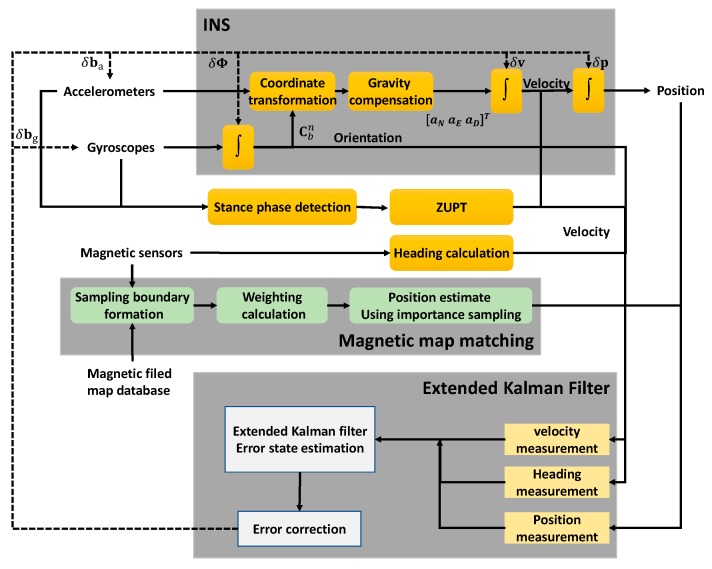
Map-matching-aided pedestrian dead reckoning (MAPDR) algorithm block diagram.

Figure 7 shows a block diagram of the proposed MAPDR algorithm. The MAPDR algorithm is roughly divided into three parts: an inertial navigation algorithm, a magnetic-field map-matching with outlier mitigation and importance sampling, and an extended Kalman filter. Basically, attitude, velocity, and position are obtained through an inertial navigation process based on the quaternion. The heading measurements from the magnetic sensor, the position measurement from the magnetic map matching, and the ZUPT are used for the EKF to correct navigation and sensor bias errors.

## 5. Experimental Results

In this study, several experiments were conducted to validate the proposed algorithm. In the experiments, MEMS-based IMUs by Xsens Inc. were used. Their specifications are listed in Table 3.

Two IMUs were mounted on the heel of a piece of footwear and a waistband buckle, respectively. One sensor was attached 5 cm above the ground, and another was attached 1 m above the ground. All sensor data were collected at a 100 Hz frequency, and the measured sensor data were received by a computer using a wireless data-receiving system by Xsens.

**Figure 8:**
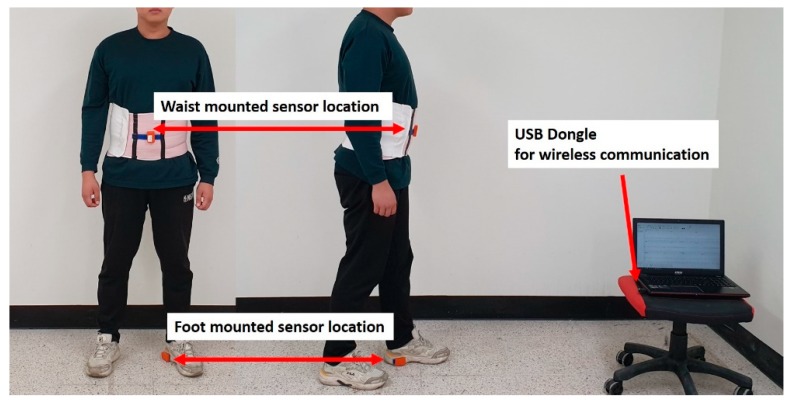
Mounted-sensors’ locations and configuration of the experiment.

The experiment sites were selected carefully to reflect many different cases. The experiments were conducted in two different buildings in Sejong University and in the different sites, such as huge halls, corridors, offices, elevator halls, and escalator halls, which have different magnetic environments. The configuration of magnetic sensors for the map building was the same as for the pedestrian experiments. The experiments were done by seven male and female experimenters with different ages. For composing a magnetic-field map, a grid size was set to 30 cm × 30 cm each. The Experimental system configuration is shown in Figure 8.

### 5.1. Experiments of Outlier Mitigation with Multi-Sensors and Roughness Weighting

**Figure 9:**
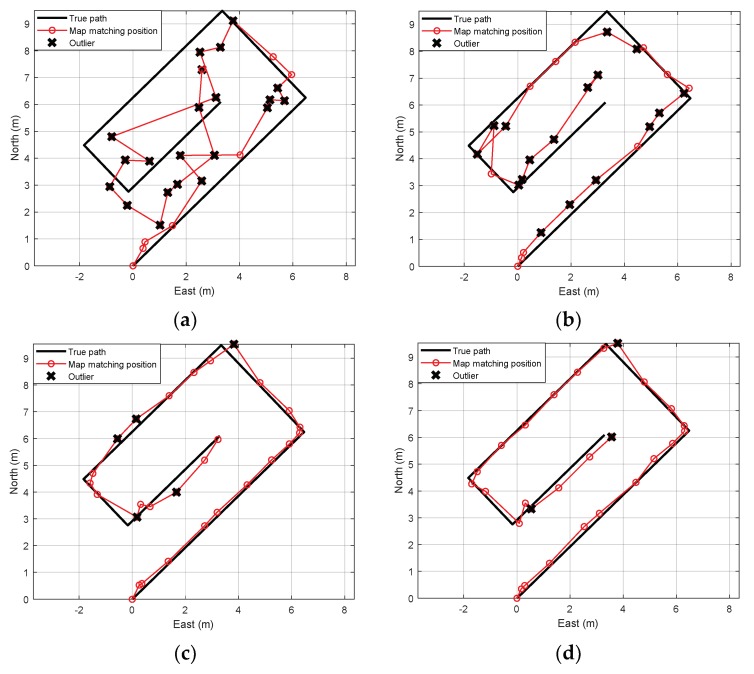
Comparison of map-matching results based on true path. (**a**) Foot-mounted single sensor case, (**b**) waist-mounted single sensor case, (**c**) multiple sensors without outlier mitigation, and (**d**) multiple sensors with outlier mitigation.

The proposed algorithm was applied, and experiments were conducted to confirm the outlier mitigation. The comparative results are shown in Figure 9, where outliers are displayed. As shown in Figure 9a,b, the position errors in cases involving the single sensor are largely due to the ambiguity and redundancy. As shown in Figure 9c, when using multiple sensors, although the error is greatly reduced and the overall path can be identified, the outliers still exist. When applying outlier mitigation with normalization, roughness weighting, and importance sampling, outliers can be reduced more significantly.

Several experimental results conducted at different sites are summarized in Table 4. Cases 1 through 4 were conducted in huge halls with different magnetic disturbance sources, such as escalators, elevators, iron doors, and iron pillars. Experiments 5 and 6 were conducted in the corridors and offices. The results in Table 4 show that the proposed algorithm mitigates outliers dramatically for all cases. For example, 48.7% of map-matched positions are outliers in the case of the single foot-mounted sensors. The outlier rate is reduced to 8.2% when the proposed mitigation algorithm is applied. 

### 5.2. Experiments of Map-Matching-Aided Pedestrian Dead Reckoning

To verify the performance of the MAPDR with the outlier mitigation algorithm using robustness weighting, normalized magnetic fields, and importance weightings, we carried out several experiments with different trajectories and different environments. The experiments were conducted repeatedly by several persons at different sites. 

**Figure 10:**
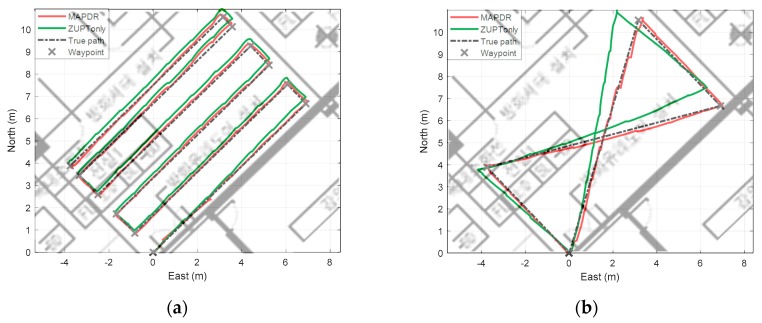

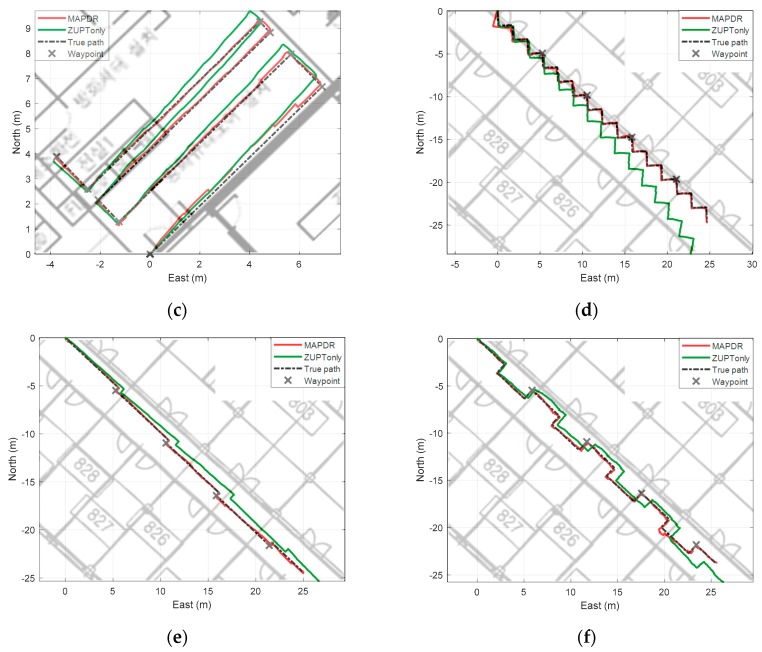
Experiment with MAPDR on the magnetic map (**a**) case 1 (**b**) case 2 (**c**) case 3 (**d**) case 4 (**e**) case 5 (**f**) case 6.

The proposed MAPDR algorithm was compared with PDR using ZUPT only, which is a conventional method, and MAPDR using a single sensor. To compare the accuracy, the average RMS errors at waypoint were compared. The critical points where the direction changes are chosen as waypoints. Each experimental result is shown in Figure 10 and RMS waypoint position errors (WPEs) are summarized in Table 5.

In the case of conventional indoor PDRs using only ZUPT, the position error is not corrected, and the position error diverges with time, which is mainly caused by heading errors and velocity errors. With MAPDR, the position is corrected every time when the stance phase starts so that an accurate position can be acquired. Moreover, the magnetic sensor attached to the waist corrects the heading, which contributes to the position accuracy. The results in Table 5 show that the proposed algorithm enhances the PDR performances effectively. 

According to the experimental results, the average RMS position error of MAPDR is less than 0.1357 m, which is smaller than the grid size of 30 cm. All of the results show that the position estimation performance is improved steadily and robustly compared with the conventional PDR using ZUPT only. 

### 5.3. Experiments of Map-Matching-Aided Pedestrian Dead Reckoning with Partial Map

In many cases, it may be difficult or sometimes impossible to build magnetic grid maps of all areas in a building. Therefore, we performed some experiments for the cases that the magnetic grid map is available partially. 

**Figure 11:**
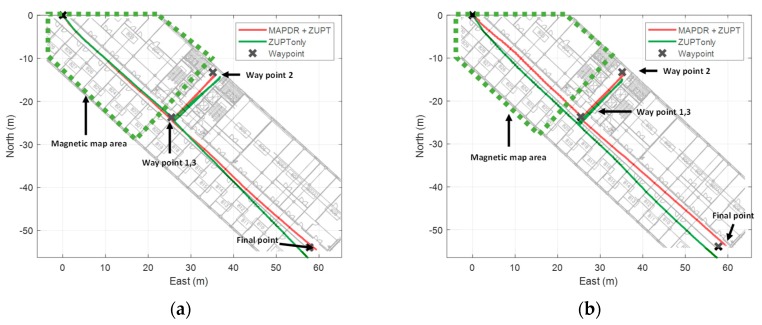
The experiment result of MAPDR with site 1 partial magnetic map (**a**) experiment 1 (**b**) experiment 2.

**Figure 12:**
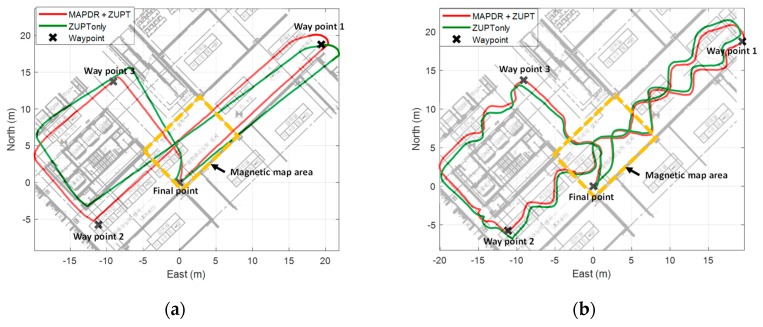
The experiment result of MAPDR with site 2 partial magnetic map (**a**) experiment 1 (**b**) experiment 2.

To verify the performance more generally, the proposed algorithm was applied to five male and female experimenters, respectively. In the area where the magnetic map is available, the position measurements from the proposed map matching with outlier mitigation were used to correct the position errors. In another area, only ZUPT was used. From the results shown in Figure 11 and Figure 12, it is confirmed that the position estimation performance is improved even when the map is partially available. Position errors in PDR using only ZUPT are mainly caused by heading and velocity errors. Especially, the initial heading error induces the position error fatally, whereas the velocity error can be compensated by the Kalman filter with ZUPT measurements. When some position measurements are available, the initial heading error as well as position error can be corrected effectively. Moreover, the magnetic information from the sensor attached to the waist contributes to the heading correction as well even though no magnetic map is available because the information is less prone to be contaminated by the external magnetic disturbances. Thus, when the pedestrian passes through the area where magnetic field map is available, the significant errors can be corrected, which results in accurate position estimation.

WPEs of all cases were compared with those of the conventional PDR, and the results are summarized in Table 6 and Table 7. We can see that the average WPEs of MAPDR at the waypoints decrease maximum by 8.372 m compared with the conventional PDR. This confirms that the proposed MAPDR reduces the position errors significantly and is also very effective, even in this case.

The results of MAPDR using a single sensor are not compared in this research. When the map matching using a single sensor is employed, the position error may not be reduced because the outliers induce incorrect estimation of navigation information, and also larger position error. In some cases, the position is estimated preposterously, which results in extreme position error more than 10 m. 

## 6. Conclusions

In this paper, we proposed a magnetic-map-matching-aided enhanced indoor PDR algorithm that uses the outlier mitigation technique. The proposed outlier mitigation algorithm is based on multiple three-axis magnetic sensors. It also employs a normalized magnetic field and roughness weighting concept for reducing the number of outliers. The importance sampling for determining the candidate-matched position is also applied to reduce the negative effects of outliers. Using the proposed mitigation algorithm, the outlier rate was drastically reduced, which resulted in the improvement of the position determination performance. Estimated positions using importance sampling were utilized as measurements for a 15th order EKF, to compensate for the navigation errors and to improve the PDR performance. 

Through numerous indoor experiments using the proposed MAPDR, we confirmed the proposed algorithm improves the overall performances. When the magnetic grid map was available in the entire walking area, the position error was significantly reduced by the position measurements. Moreover, it was confirmed that our algorithm is applicable to the area with only partial map. The performance was improved even in this case because the heading and attitude errors, as well as the position error, were corrected by the position measurements. The results imply that the cost of building a huge map database can be cut down by using the proposed algorithm. Therefore, it is concluded that an indoor MAPDR with multiple three-axis magnetic sensors and an outlier mitigation algorithm can provide a precise navigation solution compared with a conventional map-matching-based PDR using ZUPT.

To widen the application area, smartphone-based algorithm or multi-agent systems in which each agent collaborates to build a map should be studied in the future. Also, an advanced algorithm that uses another nonlinear filtering, such as point mass filter, needs to be studied. 

## Figures and Tables

**Table 1:** Comparison of the roughness in various environments.

Experiment Sites	Roughness (Mean)		
	Single Sensor (Foot, Normalized)	Single Sensor (Waist, Normalized)	Multiple Sensors (Normalized with Roughness Weighting)
Huge hall	0.5630	0.4442	0.7824
Basement hall	0.5537	0.4327	0.7805
Corridor/office	0.3335	0.2717	0.4747
Narrow corridor	0.2827	0.2923	0.4468
Average	0.4332	0.3602	0.6211

**Table 2:** Comparison of number of outliers and average position error due to outliers.

Experiment Sites	Number of Outliers (%)/Average Position Error (m)			
	Single Sensor (Foot)	Single Sensor (Waist)	Multiple Sensors	Multiple Sensors (Proposed Method)
Huge hall	88/1.9052	78/1.3411	46/0.7017	35/0.5087
Basement hall	87/2.0418	79/1.2413	34/0.4574	25/0.3951
Corridor/office	66/0.8890	81/0.8749	27/0.4184	29/0.3123
Narrow corridor	72/1.6848	76/7.6941	38/0.9190	30/0.2939
Average	78.25/1.6302	78.5/2.7879	36.25/ 0.6241	29.75/0.3775

**Table 3:** Sensor specification (www.xsens.com).

Performance Index	Gyroscope	Accelerometer	Magnetic Sensor
Full Scale	±1200°/s	$\pm 160\;m/s^{2}$	±1.5 Gauss
Non-linearity	0.1% of FS	0.2% of FS	0.2% of FS
Bias stability	10°/h	0.1 mg	-
Noise	0.05${}^{\circ}/s/\sqrt{\mathrm{Hz}}$	0.001 $m/s^{2}/\sqrt{\mathrm{Hz}}$	0.15 $\mathrm{mGauss}/\sqrt{\mathrm{Hz}}$
Alignment error	0.1°	0.1°	0.1°
Bandwidth (max)	150 Hz	150 Hz	60 Hz

**Table 4:** Comparison of outlier rates.

Experiment Cases	Outlier Rate (%)			
	Single Sensor (Foot)	Single Sensor (Waist)	Multiple Sensors	Multiple Sensors (Proposed Mitigation Algorithm)
Case1	72%	53%	17%	10%
Case2	69%	39%	18%	16%
Case3	57%	53%	34%	11%
Case4	64%	55%	16%	10%
Case5	8%	14%	4%	1%
Case6	22%	31%	9%	1%
Average	48.7%	40.8%	16.3%	8.2%

**Table 5:** Comparison of waypoint position errors.

Experiments Cases	Averaged RMS Waypoint Position Error (m)				
	Only ZUPT	MAPDR (Foot)	MAPDR (Waist)	MAPDR (Multiple Sensors Only)	MAPDR (Multiple Sensors and Proposed Mitigation Algorithm)
Case1	0.4192	1.0779	0.3232	0.2432	0.2066
Case2	0.8317	1.1690	0.6625	0.2698	0.1354
Case3	0.4098	0.8623	0.3571	0.3650	0.1489
Case4	0.8057	0.2388	1.5183	0.1701	0.1079
Case5	0.6611	0.2704	0.4728	0.1538	0.1119
Case6	0.6229	0.1722	0.2734	0.1743	0.1034
Average	0.6251	0.6318	0.6012	0.2293	0.1357

**Table 6:** Comparison of MAPDR and conventional PDR case of the partial map in experiment site 1.

WP Error (m)	WP1	WP2	WP3	Final Point
ZUPT				
Experiment1	3.0920	2.9893	2.7253	4.8752
Experiment2	2.7125	2.7832	2.5792	4.5296
Experiment3	5.4390	5.3150	5.2726	1.0671
Experiment4	5.2195	5.7721	5.0515	22.7618
Experiment5	3.6298	4.3591	3.5116	17.2025
Average WPE	4.0185	4.2437	3.828	10.0872
MAPDR (multiple sensors with proposed mitigation algorithm)				
Experiment1	0.6692	0.5664	0.4502	1.4889
Experiment2	1.4451	1.2921	1.1860	1.8411
Experiment3	2.9555	2.5820	2.4680	2.0782
Experiment4	2.2792	2.3925	1.4229	1.1447
Experiment5	1.4309	0.7774	1.0206	2.0233
Average WPE	1.7559	1.5221	1.3095	1.7152

**Table 7:** Comparison of MAPDR and conventional PDR case of the partial map in experiment site 2.

WP Error (m)	WP1	WP2	WP3	Final Point
ZUPT				
Experiment1	2.7003	2.9432	2.8493	0.9796
Experiment2	0.8682	1.1643	1.0171	0.4274
Experiment3	1.0791	1.2042	0.9999	0.5874
Experiment4	0.6080	0.3561	0.1365	0.5569
Experiment5	1.5281	1.7278	1.5468	0.8070
Average WPE	1.3567	1.4791	1.3099	0.6716
MAPDR (multiple sensors with proposed mitigation algorithm)				
Experiment1	0.6917	0.8201	0.7929	0.6918
Experiment2	0.2385	0.2712	0.2281	0.1457
Experiment3	1.0264	0.7576	0.7223	0.3101
Experiment4	0.5258	0.6093	0.2902	0.1497
Experiment5	0.2406	0.2024	0.3948	0.3385
Average WPE	0.5446	0.5321	0.4856	0.3271
